# Impact of the COVID-19 Prioritization Recommendations on Pathological Stages of Urologic Malignancies: A Real-World Analysis at a High-Volume Referral Institution

**DOI:** 10.3390/jcm13195992

**Published:** 2024-10-08

**Authors:** Antonio Andrea Grosso, Riccardo Campi, Fabrizio Di Maida, Alessio Pecoraro, Francesco Lupo Conte, Vincenzo Cangemi, Rossella Catanzaro, Neliana Kucuku, Nassima Doumit, Andrea Mari, Lorenzo Masieri, Sergio Serni, Andrea Minervini

**Affiliations:** 1 Unit of Oncologic Minimally-Invasive Urology and Andrology, Department of Experimental and Clinical Medicine, University of Florence, Careggi Hospital, 50141 Florence, Italy; fabridima90@gmail.com (F.D.M.); francescolupo.conte@unifi.it (F.L.C.); vincenzo.cangemi@unifi.it (V.C.); rossella.catanzaro@unifi.it (R.C.); neliana.kucuku@unifi.it (N.K.); nassima.doumit@unifi.it (N.D.); andrea.mari@unifi.it (A.M.); andreamine@libero.it (A.M.); 2 Unit of Urological Robotic Surgery and Renal Transplantation, Department of Experimental and Clinical Medicine, University of Florence, Careggi Hospital, 50141 Florence, Italyalessio.pecoraro10@gmail.com (A.P.); lorenzo.masieri@unifi.it (L.M.); sergio.serni@unifi.it (S.S.)

**Keywords:** oncology, COVID-19, pandemic, healthcare

## Abstract

**Background:** In response to the COVID-19 pandemic, the European Association of Urology (EAU) Guidelines defined priority groups to guide the prioritization of surgery for urological malignancies. The objective of this study was to evaluate the impact of the COVID-19 prioritization recommendations on tumor pathological characteristics in a real-world setting at our academic referral institution. **Methods:** We compared baseline and pathological tumor features of all patients with urological malignancies treated during the pandemic period (2020–2021) versus in the post-pandemic period (2022–2023). Our institution adhered to the international recommendations and prioritized those cases defined as “high-risk”. **Results:** Data from 9196 patients treated for urological malignancies were reviewed and grouped according to period of surgery (4401 in the pandemic period vs. 4785 in the post-pandemic period). The overall number of surgical procedures was comparable for all diseases except for the number of radical prostatectomies (1117 vs. 1405; *p* = 0.03) and partial nephrectomies (609 vs. 759; *p* = 0.02), which were significantly lower in the pandemic period. Regarding tumor pathological features, none of the recorded variables were found to differ according to period of surgery, including disease stage, tumor grading, presence of necrosis, lymphovascular invasion, and histological variants. **Conclusions:** A correct policy of prioritization of oncological pathologies during emergency periods and a centralization of oncological cases in reference centers reduce the possible risk of worsening cancer disease features related to the reorganization of healthcare resources.

## 1. Introduction

The COVID-19 pandemic constituted a global health crisis, significantly impacting numerous countries, including Italy, which faced an urgent necessity to reorganize its healthcare system to optimize resource allocation. The field of urology was notably affected, necessitating adjustments in both outpatient and inpatient care, with a particular emphasis on the prioritization of surgical procedures [1,2]. In response, urology centers were compelled to prioritize surgical interventions for cancer patients, implementing restrictions on elective procedures to maximize healthcare resources and minimize the risk of nosocomial infections [3,4]. Consequently, the International Associations of Urology swiftly recommended a prioritization framework for urologic surgeries, considering factors such as disease aggressiveness, the impact of short-term delays on patient outcomes, and the availability of alternative treatments [5,6]. High-priority cases that were identified included patients with bladder cancer (BCa) requiring transurethral resection (TUR-B) or radical cystectomy (RC), upper-tract urothelial carcinoma (UTUC) necessitating radical nephroureterectomy (RNU), locally advanced prostate cancer (PCa) requiring radical prostatectomy (RARP), renal cell carcinoma (RCC) involving radical nephrectomy (RN), testicular cancer necessitating orchiectomy, and penile cancer requiring penectomy. Our institution experienced operational constraints during the critical pandemic period (2020–2021) but adhered to international and governmental guidelines for prioritizing urological cancer surgeries. Nevertheless, delays were observed, particularly among patients at heightened risk of COVID-19 infection and those requiring temporary isolation due to infection. The objective of this study was to evaluate the impact of the COVID-19 prioritization recommendations on tumor pathological characteristics in a real-world setting by comparing patients treated during the pandemic period (2020–2021) with those treated in the post-pandemic period (2022–2023) at our academic referral institution.

## 2. Materials and Methods

### 2.1. Patient Data and Data Collection

All data from patients with urology cancer who were treated in an elective surgical setting at our institution (Careggi Hospital, Florence, Italy) from 2020 to 2023 were retrospectively reviewed. In detail, we collected baseline data and tumor-related features from patients who underwent RARP for PCa, TURB, RC for BCa, RNU for high-risk UTUC, partial nephrectomy (PN), or RN for renal cancer. Surgeries were grouped according to the time period of execution, in particular, during the COVID-19 pandemic (2020–2021) and post-COVID-19 pandemic (2022–2023) periods. The time frame assignment of each cohort was set in accordance with the Italian national lockdown restrictions. Patient demographic data including gender, age at surgery, Charlson comorbidity index (CCI) [7], American Society of Anesthesiologists (ASA) physical status score [8], and specific disease-related baseline features such as presence of symptoms, tumor diameter and/or complexity, and serum levels of prostate-specific antigen (PSA) for PCa patients were recorded. Tumors were classified according to the prognostic AJCC tumor, lymph node, and metastasis (TNM) staging system [9], ISUP 2016 grading system for bladder and prostate [10,11], and Fuhrman Nuclear Grade for renal carcinoma based on cellular appearance [12]. We excluded from the analysis cases having a definitive benign histotype and a palliative indication.

### 2.2. Statistical Analysis

Categorical variables were reported as absolute numbers and proportions, while continuous variables were reported as medians with interquartile ranges (IQR) or means with standard deviations (SDs) when appropriate. Chi-square and Mann-Whitney U-tests were performed for categorical and continuous variables to compare the populations, respectively. Statistical analyses were performed using STATA 13 (Stata Corp., College Station, TX, USA). All tests were two-sided, and *p* < 0.05 was considered statistically significant.

## 3. Results

In this study, data from 9196 patients treated for urological malignancies were reviewed and grouped according to period of surgery (pandemic vs. post-pandemic period). The overall number of surgical procedures was slightly lower during the pandemic period, although the difference was not significant (4401 vs. 4785). Only radical prostatectomy (1117 vs. 1405; *p* = 0.03) and partial nephrectomy (609 vs. 759; *p* = 0.02) had significant reductions during the global pandemic. Baseline characteristics of patients and tumors are reported in Table 1.

After stratifying for surgical procedure, no differences were found regarding patients’ age and sex as well as their ASA score and comorbidity burden. Tumor features and clinical presentation were also comparable between the two periods; however, for median PSA level, the number of patients undergoing radical prostatectomy was considerably higher during the global pandemic (median 10 [IQR 6–14] vs. 7 [IQR 5–13]; *p* = 0.02). Table 2 presents the tumor pathological features including stage, grading, presence of necrosis, lymphovascular invasion, and histological variants. None of the recorded variables were found to differ according to period of surgery.

## 4. Discussion

This study aims to assess the impact of the COVID-19 pandemic and the subsequent prioritization recommendations on the stage and grade of genitourinary malignancies at our institution. Numerous reports have evaluated the pandemic’s effect on surgical volumes across various cancer types [13,14]. In urologic oncology, many researchers have reported a reduction in elective oncological surgeries and an increase in surgical wait times [15,16]. This decline is attributable to the healthcare system’s burden, pandemic-induced fears, public reluctance, social isolation, and quarantine measures. At our center, we adhered to the International Guidelines proposed by the European Association of Urology (EAU) panel [6], prioritizing high-risk oncological surgeries. We observed no significant change in the total number of surgical procedures for oncological diseases, except for a reduction in RARP (1117 vs. 1405 in the pandemic and post-pandemic periods, respectively, *p* = 0.03) and PN (609 vs. 759 in the pandemic and post-pandemic periods, respectively, *p* = 0.02). This reduction may be due to the increased acceptance of alternative therapeutic strategies such as active surveillance in patients with low-risk prostate cancer (PCa) and small renal masses. Conversely, we continued to offer surgical treatment with curative intent to patients with suspected aggressive PCa and localized renal cell carcinoma (RCC), as evidenced by the higher median prostate-specific antigen (PSA) levels among PCa patients during the COVID-19 period [10 (6–14) vs. 7 (5–13) ng/mL]. On the other hand, we did not notice a significant variation in the number of surgical procedures conducted for urothelial cancer patients, as well as for those with testicular or penile cancer, which often require rapid surgical intervention that cannot be postponed. The consistent volume of elective surgical activity at our institution can be attributed to the institution’s role as a referral center, where urgent and oncologic procedures were prioritized even during the pandemic. This finding aligns with other studies conducted at referral institutions [17], while a systematic review indicated that low-volume centers experienced negative impacts on surgical delays and outcomes [18,19]. Additionally, we examined potential variations in tumor grade and stage between the pandemic and post-pandemic periods. Our analysis revealed no significant differences in pathological stage, tumor grade, or other indicators of disease aggressiveness (e.g., lymphovascular invasion, tumor necrosis, and histological variants). These findings underscore the effectiveness of the International Guidelines on surgical activity prioritization, demonstrating that, when carefully followed, they provide a valuable framework for guiding clinical and surgical practices during emergent situations [20]. The EAU Guidelines on surgical prioritization during the COVID-19 pandemic have been validated as independent predictors of oncological outcomes across different disease contexts [21,22,23]. In alignment with these guidelines, we endeavored to maintain a waiting period of 30 days for patients requiring RC, RN, RNU, orchiectomy, and penectomy while prioritizing candidates for RARP, PN, and TURBT based on imaging and baseline disease features, particularly those with suspected high-risk tumors. Although delays were inevitable in some cases, existing scientific evidence supports our approach, as several studies have highlighted that PCa, localized RCC, and superficial BCa can generally be deferred for 1 to 3 months without compromising survival outcomes [24,25].

This study has certain limitations. Primarily, it provides a snapshot of a single-center experience, which may not be generalizable to other healthcare settings. Furthermore, we were unable to assess alternative treatment modalities employed during the pandemic. Additionally, we did not compare the median duration of waiting lists for urological malignancies during the pandemic versus the post-pandemic period, limiting our ability to evaluate the impact of such delays on clinical outcomes.

Despite these limitations, our study offers a robust real-world analysis of the impact of prioritization recommendations on elective surgical activities during the COVID-19 pandemic at an academic referral institution, as well as an evaluation of potential variations in tumor stage and grade across a large patient population.

## 5. Conclusions

The present study showed that in an academic referral institution, adhering to international recommendations for prioritizing cases of “high-risk” malignancies resulted in no worsening of tumor pathological features for all urological malignancies. Centralization through referral centers and proper prioritization strategies are key to face emerging scenarios.

## Figures and Tables

**Table 1:** Baseline features of patients and diseases stratified based on period of surgery.

			Pandemic Period (2020–2021) n = 4401	Post-Pandemic Period (2022–2023) n = 4785	*p*-Value
			1117	1405	0.03
PROSTATECTOMY (n = 2522)	Age, median (IQR); years		66 (60–71)	68 (60–71)	0.11
	ASA score, median (IQR)		2 (2–2)	2 (2–3)	0.06
	CCI score, median (IQR)		3 (3–4)	3 (3–4)	0.09
	PSA serum level, median (IQR); ng/mL		10 (6–14)	7 (5–13)	0.02
			1861	1890	0.21
TURBT (n = 3751)	Gender, n. (%)	Male	1144 (61.5)	1192 (63.1)	0.21
		Female	717 (38.5)	698 (36.9)	
	Age, median (IQR); years		71 (64–78)	70 (65–77)	0.28
	ASA score, median (IQR)		2 (2–3)	2 (2–3)	0.11
	CCI score, median (IQR)		4 (3–4)	4 (3–4)	0.24
			252	246	0.18
CISTECTOMY (n = 508)	Gender, n. (%)	Male	173 (68.7)	163 (66.4)	0.33
		Female	79 (31.3)	83 (33.6)	
	Age, median (IQR); years		73 (66–78)	74 (66–78)	0.18
	ASA score, median (IQR)		2 (2–3)	2 (2–3)	0.11
	CCI score, median (IQR)		4 (3–4)	4 (3–4)	0.24
			170	185	0.34
NEPHROURETERECTOMY (n = 355)	Gender, n. (%)	Male	121 (71.4)	127 (68.8)	0.57
		Female	49 (28.6)	58 (31.2)	
	Age, median (IQR); years		70 (62–70)	70 (60–68)	0.43
	ASA score, median (IQR)		2 (2–3)	2 (2–3)	0.48
	CCI score, median (IQR)		2 (2–3)	2 (2–3)	0.33
	Hydronephrosis, n. %		89 (52.5)	93 (50.1)	0.17
			190	201	0.24
RADICAL NEPHRECTOMY (n = 391)	Gender, n. (%)	Male	113 (59.5)	116 (58.0)	0.33
		Female	77 (40.5)	85 (42.0)	
	Age, median (IQR); years		60 (52–68)	61 (52–68)	0.41
	ASA score, median (IQR)		2 (2–3)	2 (2–3)	0.38
	CCI score, median (IQR)		3 (3–4)	3 (3–4)	0.22
	Symptoms, n. (%)		44 (23.2)	38 (19.1)	0.07
			609	759	0.02
PARTIAL NEPHRECTOMY (n = 1468)	Gender, n. (%)	Male	352 (57.7)	447 (59.0)	0.51
		Female	257 (42.3)	312 (41.0)	
	Age, median (IQR); years		61 (50–67)	63 (52–68)	0.24
	ASA score, median (IQR)		2 (2–2)	2 (2–2)	0.31
	CCI score, median (IQR)		2 (2–3)	2 (2–3)	0.32
	PADUA score, median (IQR)		8 (7–9)	8 (7–9)	0.29
			86	89	0.33
ORCHIECTOMY (n = 175)	Age, median (IQR); years		34 (25–42)	36 (26–44)	0.41
	ASA score, median (IQR)		1 (1–1)	1 (1–1)	0.28
	CCI score, median (IQR)		0 (0–0)	0 (0–0)	0.24
	Tumor diameter, median (IQR); mm		2.1 (0.8–2.8)	1.9 (0.6–2.8)	0.33
			16	10	0.28
PENECTOMY (n = 26)	Age, median (IQR); years		75 (72–78)	76 (68–77)	0.18
	ASA score, median (IQR)		3 (2–3)	3 (2–3)	0.22
	CCI score, median (IQR)		4 (3–4)	4 (3–4)	0.28

ASA: American Society of Anesthesiology; CCI: Charlson Comorbidity Index; PSA: Prostate-Specific Antigen.

**Table 2:** Pathological tumor features stratified for period of surgery.

				Pandemic Period (2020–2021) n = 4401	Post-Pandemic Period (2022–2023) n = 4785	*p*-Value
				1117	1405	0.03
PROSTATECTOMY (n = 2522)	pT stage, n. (%)	pT2		424 (37.9)	542 (38.5)	0.18
		pT3a		511 (45.7)	602 (43.0)	
		pT3b		177 (16.0)	254 (18.0)	
		pT4		5 (0.4)	7 (0.5)	
	pN+, n. (%)			159 (14.2)	210 (14.9)	0.24
	ISUP grade, median (IQR)			3 (2–4)	3 (2–4)	0.33
				1861	1890	0.21
TURBT (n = 3751)	pT stage, n. (%)		pTis	53 (2.8)	58 (3.1)	0.14
			pTa	786 (42.2)	776 (41.1)	
			pT1	893 (47.9)	871 (46.0)	
			pT2	129 (7.1)	185 (9.8)	
	Tumor grade, n. (%)		Low	998 (53.6)	1022 (54.0)	0.38
			High	863 (46,4)	868 (46.0)	
	Concomitant CIS, n. (%)			241 (12.9)	205 (10.8)	0.29
				252	246	0.18
CISTECTOMY (n = 508)	pT stage, n. (%)		pT2	105 (41.6)	111 (45.1)	0.41
			pT3a	95 (37.6)	89 (36.1)	
			pT3b	45 (18.1)	37 (15.2)	
			pT4	7 (2.7)	9 (3.6)	
	pN+, n. (%)			41 (16.2)	38 (15.4)	0.38
	Histology variant, n. (%)			32 (12.6)	32 (13.0)	0.28
				170	185	0.34
NEPHROURETERECTOMY (n = 355)	pT stage, n. (%)		pT1	43 (25.2)	39 (21.0)	0.17
			pT2	96 (56.4)	109 (58.9)	
			pT3	29 (17.0)	33 (18.0)	
			pT4	2 (1.4)	4 (2.1)	
	pN+, n. (%)			25 (14.7)	31 (16.7)	0.24
	Lymphovascular invasion, n. (%)			53 (25.2)	46 (24.8)	0.28
	Histology variant, n. (%)			19 (11.1)	17 (9.1)	0.45
				190	201	0.24
RADICAL NEPHRECTOMY (n = 391)	pT stage, n. (%)		pT1b	24 (12.6)	27 (13.4)	0.19
			pT2	69 (36.3)	70 (34.8)	
			pT3a	81 (42.6)	89 (44.2)	
			pT3b/c	10 (5.2)	13 (6.4)	
			pT4	6 (3.3)	2 (1.2)	
	pN+, n. (%)			40 (21.0)	38 (18.9)	0.31
	Nuclear grade, median (IQR)			3 (3–4)	3 (3–4)	
	Necrosis, n. (%)			64 (33.6)	72 (35.8)	0.22
				609	759	0.02
PARTIAL NEPHRECTOMY (n = 1468)	pT stage, n. (%)		pT1a	339 (55.6)	401 (52.8)	0.44
			pT1b	158 (25.9)	221 (29.1)	
			pT2	28 (4.5)	36 (4.8)	
			pT3a	84 (14.0)	101 (13.3)	
	Nuclear grade, median (IQR)			2 (1–3)	2 (1–3)	0.19
	Necrosis, n. (%)			93 (13.1)	101 (13.3)	0.56
				86	89	0.33
ORCHIECTOMY (n = 175)	Disease stage, n. (%)	Ia		19 (22.0)	17 (19.1)	0.26
		Ib		18 (22.1)	22 (24.7)	
		Is		20 (23.2)	15 (16.8)	
		II		21 (23.4)	26 (29.3)	
		III		8 (9.3)	9 (10.1)	
	Lymphovascular invasion, n. (%)			15 (17.4)	17 (19.1)	0.19
	Non-seminomatous germ cell tumors, n. (%)			39 (45.3)	38 (42.6)	0.22
				16	10	0.28
PENECTOMY (n = 26)	Disease stage, n. (%)	I		4 (25.0)	3 (30.0)	0.37
		IIa		5 (31.2)	4 (40.0)	
		IIb		3 (18.7)	2 (20.0)	
		IIIa		3 (18.7)	1 (10.0)	
		IIIb		1 (6.4)	0 (0.0)	
		IV		0	0 (0.0)	
	Histopathological grading, median, (IQR)			2 (1–3)	2 (1–3)	0.22

## Data Availability

Data are available on request due to restrictions (e.g., privacy or ethical).
